# Religion or class? Measuring voting clustering on religious and socioeconomic lines in US presidential elections

**DOI:** 10.1371/journal.pone.0331959

**Published:** 2025-10-06

**Authors:** Julián Villaseñor-Ibáñez, Marcelo del Castillo-Mussot, Omar El Deeb

**Affiliations:** 1 Intituto de Fisica, Universidad Nacional Autónoma de México, México City, México; 2 Mathematics Department, University of Warwick, Coventry, United Kingdom; Universidade Estadual de Maringa, BRAZIL

## Abstract

Electoral behavior in the United States is shaped by more than geography and economics as it is deeply intertwined with cultural identity. Here, we quantify how voting patterns in the 2016, 2020 and 2024 presidential elections cluster not only across neighboring counties but also along shared religious and socioeconomic lines. By computing Moran’s I, a standard measure of spatial autocorrelation, under four distinct “neighborhood” definitions (physical borders, dominant religion, income bracket and urbanization level), we show that counties sharing a majority faith vote in strikingly similar ways, second only to contiguous geography. In contrast, grouping by household income or urban status yields markedly weaker clustering. These findings reveal that cultural networks, embodied by religious affiliation, exert a stronger influence on aggregate voting behavior than class differences or the urban–rural divide. Our approach highlights the power of simple network models grounded in social traits to illuminate the dynamics of political polarization and suggests new pathways for understanding how cultural identity shapes large scale electoral outcomes.

## Introduction

The role of religion in the politics of the United States remains a topic of study for social scientists [1–3]. While the legal principle of separating church and state is firmly established, the influence of faith, particularly on public discourse and electoral behavior, cannot be overlooked. Over the past decade, as demographic shifts, evolving social issues, and deepening political polarization have reshaped the American political landscape, religion has emerged as a central theme in political rhetoric, especially during the Trump era. Understanding how religious identity shapes voting behavior is not only important for analyzing recent elections but also for grasping broader cultural dynamics at play in contemporary U.S. society. It is thus crucial to devise methods that can quantify this complex and often subtle social phenomenon.

Sociophysics offers a unique lens through which to examine such societal trends by applying principles and methodologies traditionally used in the physical sciences [4–6]. This field emphasizes the use of quantitative tools to model and understand social behaviors, including patterns in voter decision-making and election outcomes. For instance, measures derived from entropy have been employed to assess uncertainty and distribution in vote shares across candidates [7–9]. Similarly, models of collective voter dynamics, accounting for interpersonal interactions, media influence, and opinion evolution, have provided valuable insights into how electoral outcomes emerge from complex social systems [10–17]. As sociophysics models increasingly map collective behavior, our work applies spatial autocorrelation to electoral data, bridging gaps between cultural identity and political outcomes. These interdisciplinary approaches enhance our ability to interpret and predict political behavior, offering tools that go beyond traditional qualitative analysis.

Elections not only determine political leadership but also reflect the collective behavior of diverse communities responding to social, economic, and cultural conditions—making them valuable for studying patterns of human decision-making and public opinion shifts over time. The election of Donald Trump in 2016 marked a turning point in American politics, ushering in a period defined by intense debates over race, class, and religion [3,18–20]. His presidency highlighted how deeply intertwined these identities had become in shaping political preferences. Research into this era has increasingly focused on partisan polarization [21], including its intensification during the global health crisis of 2020 [22]. A key area of inquiry has been the interplay between cultural or ethnic identities and socioeconomic status, particularly how these factors compete or align in influencing political choices [23–26].

Previous research has quantified substantial differences in voting preferences across religious groups; for example, Pew Research Center data for the 2020 U.S. presidential election show that White evangelical Protestants voted about 84% for the Republican candidate, while religiously unaffiliated voters supported the Democratic candidate at about 71%, patterns consistent with earlier findings of 20–30 percentage point partisan gaps between religious traditionalists and modernists [1,3,27].

By combining statistical modeling with spatial and temporal data, researchers can uncover hidden trends in electoral behavior, helping inform both academic understanding and public discourse on the evolving landscape of political participation. Given that religion, or one’s stance toward it, is a core component of personal and communal identity [28], we argue that examining its impact on electoral outcomes can reveal significant insights into the cultural forces shaping modern politics. Building on our earlier work, which introduced a quantitative analysis of religion’s influence during the 2020 election using spatial and confessional autocorrelation at the state level [29], we now extend this research to the county level. Recent studies highlight deepening cultural polarization in U.S. politics, our analysis extends this by quantifying religion’s outsized role over class in voting behavior. Understanding voting behavior at the county level offers insights into regional dynamics that national analyses may overlook, such as the influence of local demographics, historical affiliations, or community-specific issues on electoral outcomes.

We analyze voting patterns from the 2016, 2020, and 2024 U.S. presidential elections using Moran’s Index, a widely used measure of spatial clustering [30], to explore how religious, economic, and urban characteristics correlate with electoral behavior. By incorporating additional socioeconomic indicators such as median income and urbanization levels, we aim to compare the relative influence of cultural versus economic factors in shaping voting clusters.

Statistical and mathematical techniques provide an objective framework for analyzing electoral data [31]. Our primary analytical tool is spatial autocorrelation, which quantifies the degree to which similar values cluster geographically or along other defined dimensions [32]. A recent study extended Moran-type statistics to complex networks, explicitly demonstrating how network-defined weight matrices can be used instead of geographic distance, supporting our methodological choice to design weight structures based on non-spatial neighborhood relations [33]. Positive autocorrelation implies that similar voting patterns tend to group together, while negative autocorrelation suggests dissimilar outcomes cluster closely. A zero value indicates randomness. This method allows us to move beyond simple geographic proximity and explore whether shared religious affiliations or socioeconomic conditions better explain similarities in voting behavior across counties.

We structure this paper as follows: The introduction provides background and motivation; We then outline the mathematical and statistical methods we employ, including definitions of adjacency based on geography, religion, income, and urbanization. Then, we present and interpret our results, and We finally conclude with a discussion of implications and future directions.

## Mathematical and statistical methods

### Spatial autocorrelation

Everything is related to everything else, but near things are more related than distant things, an observation which has come to be referred to as Tobler’s First Law of Geography, exemplifies the notion of spatial autocorrelation. This is a statistical concept that quantifies the degree of correlation between variables in geographical space. A particular measure of this quantity is Moran’s I, which tests whether the values in global space are clustered, dispersed, or randomly distributed.

For a given variable, Moran’s I considers the case where it is randomly distributed across space, and compares it to the observed data arrangement, by taking into account the spatial relationship between locations and the values at each location. The formula calculates the covariance of every pair of values corresponding to distinct locations, weighted by the distance between them. It is given by [30]:

$$I=\frac{N\Sigma_{ij}W_{ij}(X_{i}-\bar{X})(X_{j}-\bar{X})}{\Sigma_{ij}W_{ij}\Sigma_{i}(X_{i}-\bar{X}{)}^{2}}$$ (1)

Here, *N* denotes the total number of spatial units in the dataset (in our case, the number of U.S. counties considered), *X*_*i*_ and *X*_*j*_ are the values at locations *i* and *j*, and $\bar{X}$ is their mean. $X_{i}-\bar{X}$ and $X_{j}-\bar{X}$ are defined as the deviation from the mean for each data value. The distance between values is given by *W*_*ij*_, which corresponds to the entries of a spatial weight matrix. The diagonal terms are taken to be zero *W*_*ii*_ = 0.

Moran’s I ranges from –1 to  + 1. Values close to  + 1 indicate strong positive spatial autocorrelation (that is, similar values are clustered together), while values close to –1 suggest strong negative spatial autocorrelation (dissimilar values are clustered together). Values close to 0 imply the values are spatially randomly distributed, showing no significant spatial autocorrelation.

We also define the following *z*_*I*_-score associated to these statistics:

$$z_{I}=\frac{I-E[I]}{\sqrt{V[I]}}$$ (2)

where the expected value *E*[*I*] and the variance *V*[*I*] are defined in Appendix A. The *z*-score or its associated *p*-value are used to reject the null hypothesis and the possibility of a random pattern leading to the obtained Moran *I* value. Here, we adopt a 95% confidence level of *z*_*I*_>1.96 corresponding to *p* < 0.05 in order to confirm the significance of our results.

We use Moran’s I in order to quantify the degree of spatial autocorrelation for US presidential election results (2016, 2020 and 2024) at the county level. That is, we measure the degree of similarity between the percentage of votes going to the Democratic or Republican candidate in adjacent counties. However, apart from the usual Geographical adjacency (counties are adjacent if they border each other), we further define Confessional adjacency, Median Household Income adjacency and Urbanization Level adjacency by constructing the relevant matrices. The process of defining these matrices can be summarized as assigning a category to each county in the continental US, and then defining county adjacency in terms of that category: counties neighbor those other counties in their same category. Moran’s I was calculated using the Moran.I() function from the ape package in the R statistical computing environment.

The corresponding p values in R are given as zero, but we report them as *p* < 0.001. While significance here is assessed using the analytical *z*-score formulation implemented in the Moran.I() function of the ape package in R, we note that an alternative non-parametric approach would be to fix the adjacency matrix and randomly permute the observed variable among counties; given the consistently large *I* values and extremely small *p*-values obtained in our analysis, we expect such a permutation framework would yield the same conclusions.

Comparing resulting Moran’s Is gives an idea of the influence of different factors on voting behavior: geographical, religious-cultural or economic. A higher Moran’s I for Confessional adjacency than for Median Income adjacency would show a tendency of voters to cast their votes following their religious peers (even if half a country away), rather than voting similarly to their peers belonging to the same income level.

While our primary measure of global spatial autocorrelation is Moran’s I, alternative formulations such as Lee’s statistic [34] could equally be applied to our adjacency matrices, given prior evidence of strong qualitative agreement between these measures in network structured contexts [33], we expect our main conclusions to remain robust, and we leave a detailed comparison to future work.

### Geographic adjacency matrix

We used data from the US Census Bureau 2024 County Adjacency File [35] in order to create the Geographical adjacency matrix, *W*_*ij*_, which has non-zero entries for counties bordering each other and zero for those that do not. As stated above, the diagonal terms are taken to be zero.

$$W_{ij}=\left\{ \begin{array}{cc} 1 & \text{if counties }i\text{ and }j\text{ border eachother}\\ 0 & \text{Otherwise} \end{array}\right.$$ (3)

### Confessional adjacency matrix

The 2020 Public Religion Research Institute (PRRI) Census of American Religion lists the largest religious denominations by percentage in each county [36], grouping both race and religion in single categories, owing to the important interplay between these two factors in the American political landscape. In the PRRI dataset, religious affiliation is recorded in categories that combine both denominational and racial/ethnic descriptors. Examples include *White Evangelical Protestant*, *White Catholic*, *Hispanic Catholic*, *Black Protestant*, *Other Christian*, *Jewish*, *Muslim*, *Buddhist*, *Hindu*, and *Unaffiliated* (including atheist, agnostic, and those with no religious affiliation). As discussed below, we treat these categories as distinct groups when constructing our confessional adjacency matrices, with the exception that in one variant (*First Religion (CHR)*), where CHR stands for “Christian,” indicating that all White Christian groups (e.g., White Evangelical Protestant, White Catholic, White Mainline Protestant) are grouped together as in the original PRRI dataset. For example, White Catholic is presented as a distinct category than Hispanic Catholic. With this data we assign a First, Second, Third and Fourth largest religious group for each county, which we use to construct the Confessional Majority Adjacency Matrices for each case.

However, since this census groups many confessional groups into the All White Christian classification (which is the largest category in the majority of counties), we worked with the data so that the First, Second, Third and fourth largest religion by county do not group together all White Christians: i.e. White Catholics and White Evangelical Protestants are counted separately. However, we do take into account this All White Christian grouping in order to present an additional Confessional Majority adjacency matrix that takes into account this group’s prevalence across the United States. Finally, our data set also includes important percentages of Unaffiliated Americans, comprising those not identifying with a particular religion, as well as atheists and agnostics. These are treated as any other religious category, although the Census does not make any distinction based on their ethnic origins.

To define our Confessional adjacency matrices *W*_*ij*_, for the case of the First largest religion, we take counties to be adjacent if they share the same largest religion and non-adjacent otherwise. The process is similar for the Second, Third and Fourth largest religions. Finally, we also calculate an additional First largest religion adjacency matrix in which we keep intact the All White Christian category, just like in the original data set. We denote this adjacency matrix and subsequent results deriving from it as First Religion (CHR).

**Fig 1:**
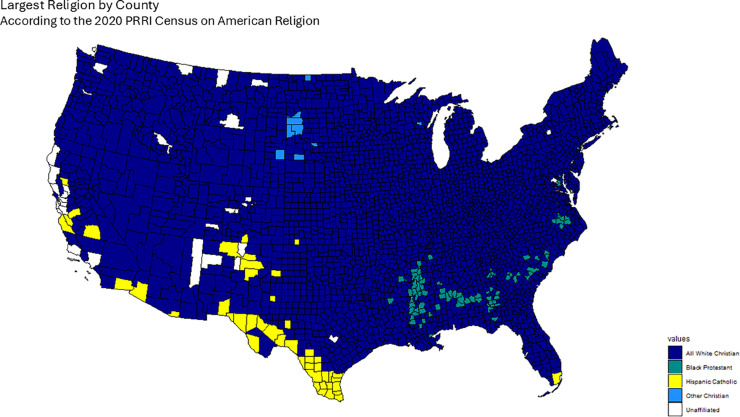
Largest religion in each county by percentage of adherents according to the PRRI. We construct the Confessional Adjacency matrix according to this data, by defining adjacency in terms of a shared dominant religious congregation.

**Fig 2:**
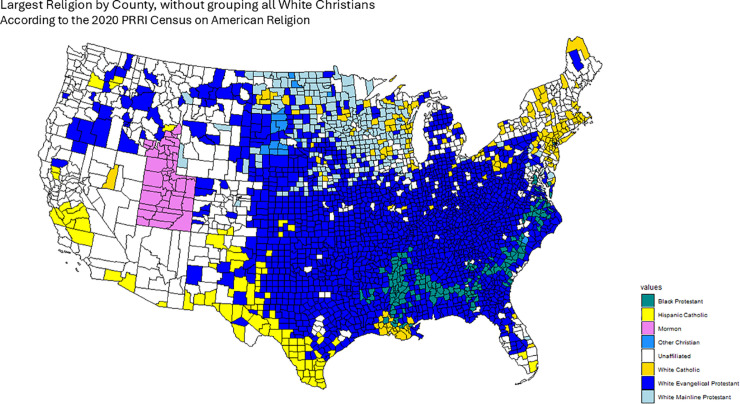
Largest religion in each county by percentage of adherents according to the PRRI. We construct the First Religion Confessional Adjacency matrix according to this data, by defining adjacency between counties if they share the same religion.

**Fig 3:**
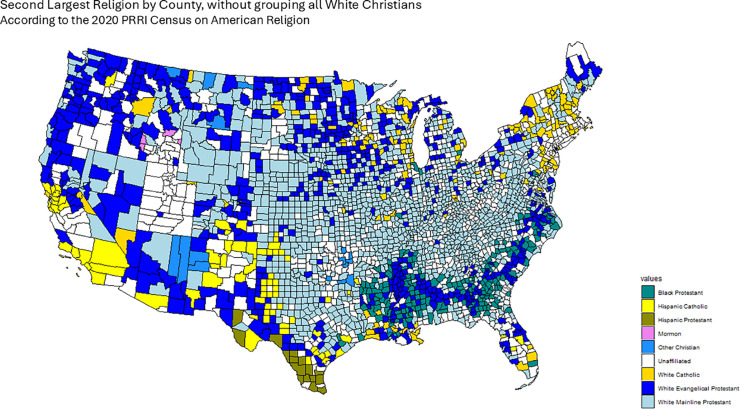
Second largest religion in each county by percentage of adherents according to the PRRI. We construct the Second Religion Confessional Adjacency matrix according to this data, by defining adjacency between counties if they share the same religion.

**Fig 4:**
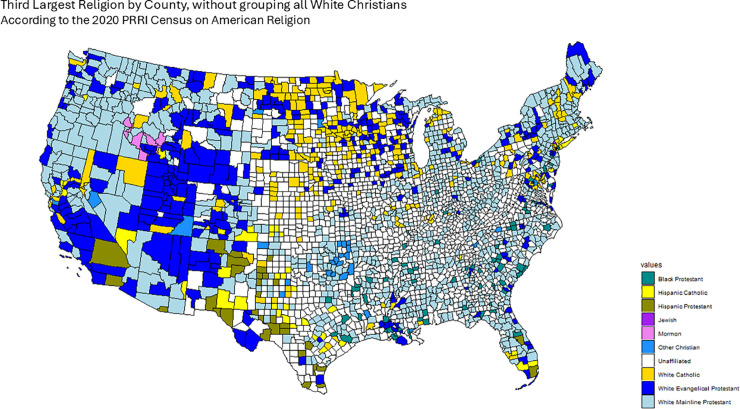
Third largest religion in each county by percentage of adherents according to the PRRI. We construct the Third Religion Confessional Adjacency matrix according to this data, by defining adjacency between counties if they share the same religion.

**Fig 5:**
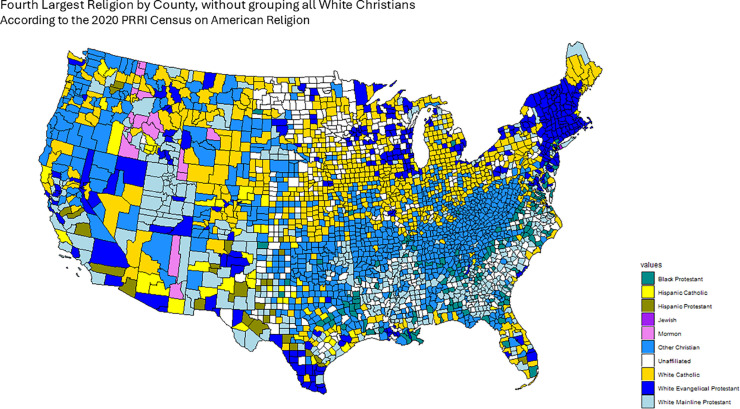
Fourth largest religion in each county by percentage of adherents according to the PRRI. We construct the Fourth Religion Confessional Adjacency matrix according to this data, by defining adjacency between counties if they share the same religion.

Figs 1, 2, 3, 4 and 5 graphically show the five cases we have discussed here. Each map corresponds to an adjacency matrix, formally defined as:

$$W_{ij}=\left\{ \begin{array}{cc} 1 & \text{if counties }i\text{ and }j\text{ have the same (First, Second, Third or Fourth) religion}\\ 0 & \text{Otherwise} \end{array}\right.$$ (4)

### Median household income adjacency

The Small Area Income and Poverty Estimates (SAIPE) program of the US Census Bureau produces yearly estimates of the median household income by county [37]. We use the 2021 estimates to group counties into one of five categories according to their median household income in USD:

- Category 1 - Median Household Income is less than $50, 000
- Category 2 - Median Household Income ranges from $50, 000 to $75, 000
- Category 3 - Median Household Income ranges from $75, 000 to $100, 000
- Category 4 - Median Household Income ranges from $100, 000 to $125, 000
- Category 5 - Median Household Income is more than $125, 000

**Fig 6:**
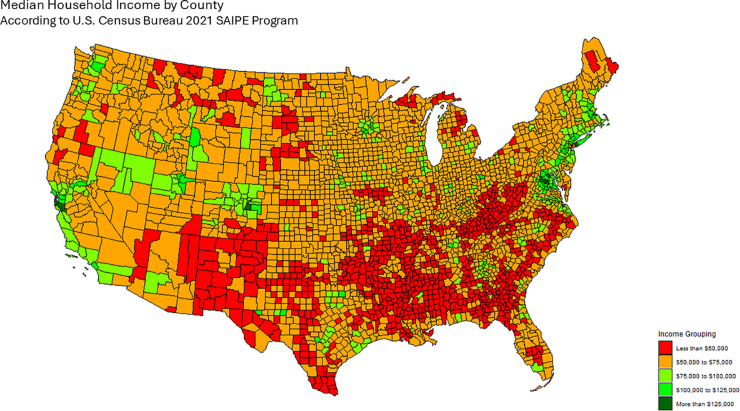
Median household income by county according to data from the SAIPE. We construct the Median Household Income Adjacency matrix according to this data, by defining adjacency between counties if they belong to the same income bracket.

The map in Fig 6 shows the category to which each county belongs. This allows us to define an Median Household Income adjacency matrix:

$$W_{ij}=\left\{ \begin{array}{cc} 1 & \text{if counties }i\text{ and }j\text{ belong to the same income grouping}\\ 0 & \text{Otherwise} \end{array}\right.$$ (5)

### Urbanization level adjacency

The 2013 National Center for Health Statistics (NCHS) Urban-Rural Classification Scheme for Counties examines data from every county in the United States, sorting them into one of five different categories according to their urbanization level and population [38]: Large Central Metro, Large Fringe Metro, Medium Metro, Small Metro, Micropolitan, and Non-Core, which in turn belong to either Metropolitan Statistical Areas (MSA) or Micropolitan Statistical Areas. A Metropolitan Statistical Area is a geographic entity based on a county or group of counties with at least one urbanized area, a population of at least 50,000 and adjacent counties with economic ties to the central area. Micropolitan Statistical Areas are defined similarly, but with an urban nucleus consisting of counties with a population between 10,000 and 49,999. The criteria used to define each category are the following:

- Large central metro : Counties in MSAs with a population of 1 million that either:
  - Contain the entire population of the largest principal city of the MSA
  - Have their entire population contained in the largest principal city of the MSA, or,
  - Contain at least 250,000 inhabitants of any principal city of the MSA
- Large fringe metro: Counties in MSAs with a population of 1 million or more that did not qualify as Large Central Metro.
- Medium metro: Counties in MSAs with a population between 250,000 and 999,999.
- Small metro: Counties in MSAs with a population less than 250,000
- Micropolitan: Counties in Micropolitan Statistical Areas
- Non-core: Counties that did not qualify for Micropolitan Statistical Areas. These are the most rural areas in the US.

**Fig 7:**
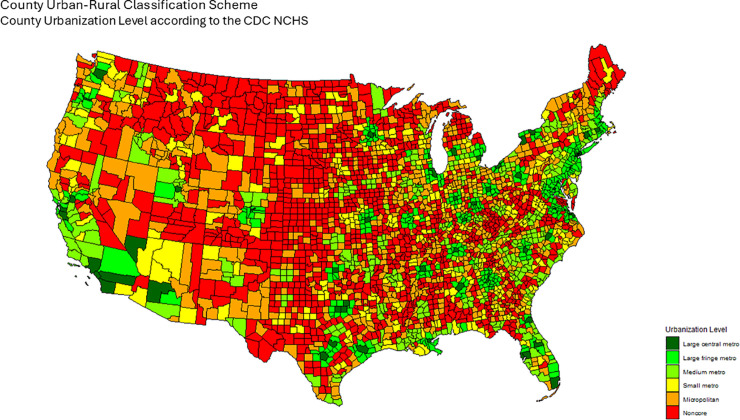
Urbanization level by county according to the NCHS classification scheme. We construct the Urbanization Level adjacency matrix according to this data, by defining adjacency between counties if they share the same urbanization level.

The map in Fig 7 shows counties according to their NCHS classification. This allows us to define the Urbanization Level adjacency matrix in the following way:

$$W_{ij}=\left\{ \begin{array}{cc} 1 & \text{if counties }i\text{ and }j\text{share the same urbanization level}\\ 0 & \text{Otherwise} \end{array}\right.$$ (6)

While more graded weighting schemes could be used (e.g., inverse-distance weights for geography or similarity scores between denominations), we are not aware of any widely accepted, objective metric that would allow robust and non-arbitrary quantification of such distances. For clarity, reproducibility, and comparability across adjacency definitions, we therefore adopt binary weight matrices throughout.

## Results and discussion

Our method allows us to compare the degree of similarity between the percentage of votes for Republican or Democratic presidential candidates in adjacent counties. By defining different types of adjacency, a Moran’s I value of  + 1 shows counties in the same category tend to vote similarly, while a value of –1 implies dissimilar voting tendencies within this same category. Values close to 0 mean a random distribution of vote percentages. Since the percentage of votes for third candidates is negligible, the percentage of votes for the Republican and Democratic candidate is mirrored across counties, resulting in very similar values in Moran’s I values.

The analysis reveals that counties sharing a majority religion exhibit strong voting pattern similarity, surpassing clustering by income or urbanization levels. Geographically adjacent counties show the highest Moran’s I (*I*_*G*_ = 0.605, *I*_*G*_ = 0.596 and *I*_*G*_ = 0.604 for the case of Trump voting percentages for the 2016, 2020 and 2024 elections, respectively) for all years. Thus, geographic adjacency remains the most decisive factor in predicting whether two counties will vote similarly.

We find that the next best predictor is the majority religion in the county (First Religion Confessional Autocorrelation), with high Moran’s I values ($I_{R_{1}}=0.506$, $I_{R_{1}}=0.493$ and $I_{R_{1}}=0.509$ for the case of Trump voting percentages for the 2016, 2020 and 2024 elections, respectively) indicating a tendency of counties of the same cultural religious identity to vote similarly, even if they do not form a continuous geographical block. This trend persisted consistently across the 2016, 2020, and 2024 elections, highlighting religion’s enduring influence on electoral behavior during the Trump era (see Tables 1, 2 and 3).

**Table 1:** Moran’s I for 2016 Trump/Clinton vote share when taking into account Geographic, Confessional (1st (CHR), 1st, 2nd, 3rd and 4th majority religion), Median Household Income and Urbanization Level Adjacency. First Religion (CHR) refers to the majority religion when grouping together all White Christian groups (see 1)

2016 Presidentiap Epections	Statistic	Trump %	Cpinton %
Geographicap Spatiap Autocorrepation	Moran Index (*I*_*G*_)	0.6048724	0.6069526
	p-vapue	<0.001	<0.001
First Repigion (CHR) Confessionap Autocorrepation	Moran Index (*I*_*R*_)	0.2585688	0.2862953
	p-vapue	<0.001	<0.001
First Repigion Confessionap Autocorrepation	Moran Index ($I_{R_{1}}$)	0.5055928	0.4971626
	p-vapue	<0.001	<0.001
Second Repigion Confessionap Autocorrepation	Moran Index ($I_{R_{2}}$)	0.01335405	0.01114453
	p-vapue	<0.001	<0.001
Third Repigion Confessionap Autocorrepation	Moran Index ($I_{R_{3}}$)	0.01247968	0.009805721
	p-vapue	<0.001	<0.001
Fourth Repigion Confessionap Autocorrepation	Moran Index ($I_{R_{4}}$)	0.01711847	0.01502573
	p-vapue	<0.001	<0.001
Income Autocorrepation	Moran Index (*I*_*I*_)	0.1283752	0.1202321
	p-vapue	<0.001	<0.001
Urbanization Autocorrepation	Moran Index (*I*_*U*_)	0.179511	0.1765446
	p-vapue	<0.001	<0.001

**Table 2:** Moran’s I for 2020 Trump/Biden vote share when taking into account Geographic, Confessional (1st (CHR), 1st, 2nd, 3rd and 4th majority religion), Median Household Income and Urbanization Level Adjacency. First Religion (CHR) refers to the majority religion when grouping together all White Christian groups (see 1)

2020 Presidentiap Epection	Statistic	Trump %	Biden %
Geographicap Spatiap Autocorrepation	Moran Index (*I*_*G*_)	0.5964326	0.5983807
	p-vapue	<0.001	<0.001
First Repigion (CHR) Confessionap Autocorrepation	Moran Index (*I*_*R*_)	0.2291959	0.2349533
	p-vapue	<0.001	<0.001
First Repigion Confessionap Autocorrepation	Moran Index ($I_{R_{1}}$)	0.492521	0.4900599
	p-vapue	<0.001	<0.001
Second Repigion Confessionap Autocorrepation	Moran Index ($I_{R_{2}}$)	0.01317262	0.01318949
	p-vapue	<0.001	<0.001
Third Repigion Confessionap Autocorrepation	Moran Index ($I_{R_{3}}$)	0.01472684	0.01420664
	p-vapue	<0.001	<0.001
Fourth Repigion Confessionap Autocorrepation	Moran Index ($I_{R_{4}}$)	0.01755783	0.01774572
	p-vapue	<0.001	<0.001
Income Autocorrepation	Moran Index (*I*_*I*_)	0.1453396	0.1430037
	p-vapue	<0.001	<0.001
Urbanization Autocorrepation	Moran Index (*I*_*U*_)	0.2022066	0.2012412
	p-vapue	<0.001	<0.001

**Table 3:** Moran’s I for 2024 Trump/Harris vote share when taking into account Geographic, Confessional (1st (CHR), 1st, 2nd, 3rd and 4th majority religion), Median Household Income and Urbanization Level Adjacency. First Religion (CHR) refers to the majority religion when grouping together all White Christian groups (see 1)

2024 Presidentiap Epections	Statistic	Trump %	Harris %
Geographicap Spatiap Autocorrepation	Moran Index (*I*_*G*_)	0.6042822	0.6034301
	p-vapue	<0.001	<0.001
First Repigion (CHR) Confessionap Autocorrepation	Moran Index (*I*_*R*_)	0.2186605	0.2212994
	p-vapue	<0.001	<0.001
First Repigion Confessionap Autocorrepation	Moran Index ($I_{R_{1}}$)	0.5091182	0.5049949
	p-vapue	<0.001	<0.001
Second Repigion Confessionap Autocorrepation	Moran Index ($I_{R_{2}}$)	0.01442632	0.01496071
	p-vapue	<0.001	<0.001
Third Repigion Confessionap Autocorrepation	Moran Index ($I_{R_{3}}$)	0.01511468	0.01430034
	p-vapue	<0.001	<0.001
Fourth Repigion Confessionap Autocorrepation	Moran Index ($I_{R_{4}}$)	0.01614826	0.01715249
	p-vapue	<0.001	<0.001
Income Autocorrepation	Moran Index (*I*_*I*_)	0.1596265	0.1563115
	p-vapue	<0.001	<0.001
Urbanization Autocorrepation	Moran Index (*I*_*U*_)	0.2047286	0.2068657
	p-vapue	<0.001	<0.001

Autocorrelation values for the second, third, and fourth largest religion in each county are close to zero. As can be seen in the accompanying maps, the distribution of these religious groups across counties appears to have a much more random character, owing to the United States’ strong religious diversity. They do not hold sway over the majority of the population, and thus can be discarded as driving forces that can prompt counties to vote in similar manners. Higher, yet still weak, autocorrelation values are obtained when, as in the original dataset, the distinction is not made between the different White Christian groups (*I*_*R*_ = 0.259, *I*_*R*_ = 0.229, *I*_*R*_ = 0.219 for the case of Trump voting percentages for the 2016, 2020 and 2024 elections, respectively). Since, in this framework, the resulting distribution of majority religion by county is an approximation of the majority ethnic group by county, this suggests a high degree of diversity in the political leanings of White Americans according to their religious identities.

Defining adjacency in terms of socioeconomic factors also yields positive but weak Moran’s I values. Income autocorrelation values (*I*_*I*_ = 0.128, *I*_*I*_ = 0.145, *I*_*I*_ = 0.160 for the case of Trump voting percentages for the 2016, 2020 and 2024 elections, respectively) do not speak of very strong shared voting patterns across counties in the same bracket. Urbanization is a slightly better predictor of similar county electoral behavior (*I*_*U*_ = 0.180, *I*_*U*_ = 0.202, *I*_*U*_ = 0.205), but these values still fall short of those corresponding to First Religion Autocorrelation. The degree of similarity between voting patterns across counties is highest for Geographical adjacency, then First Religion adjacency, First Religion (CHR) adjacency, Urbanization adjacency, Income adjacency and finally minority religions adjacency (Second, Third and Fourth).

We note that the adjacency definitions used here may not be statistically independent: in the United States, majority-religion patterns, income levels, and urbanization each exhibit geographic clustering, leading to partial overlap between these matrices. Our analysis treats each framework separately, and the consistently higher Moran’s I for First Religion relative to income or urbanization suggests that religious identity remains a meaningful predictor of voting similarity even when some correlation with geography is expected.

**Fig 8:**
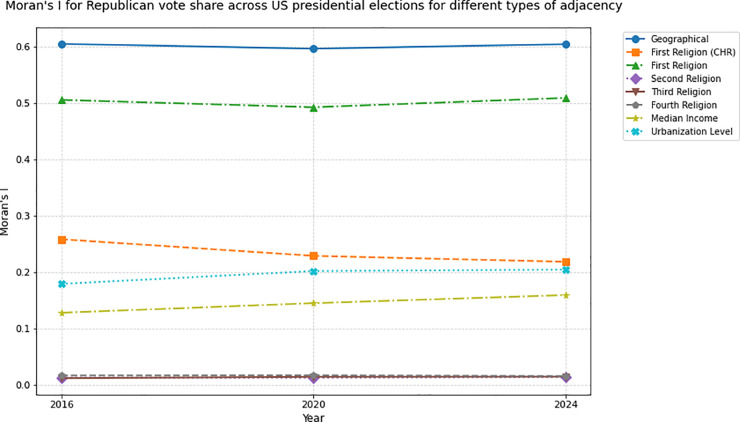
Moran’s I for Republican share of total votes across US presidential elections, under different adjacency frameworks: Geographical, First Religion (CHR), First Religion, Second Religion, Third Religion, Fourth Religion, Median Income, Urbanization Level. Time variance is minimal, order among adjacency types is preserved.

**Fig 9:**
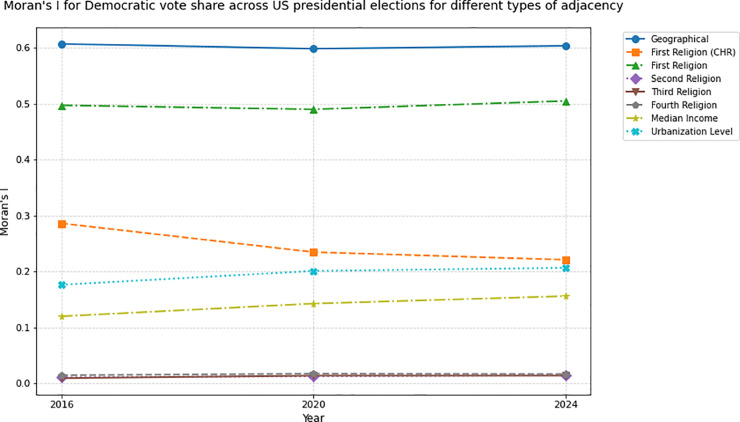
Moran’s I for Democratic share of total votes across US presidential elections, under different adjacency frameworks: Geographical, First Religion (CHR), First Religion, Second Religion, Third Religion, Fourth Religion, Median Income, Urbanization Level. Time variance is minimal, order among adjacency types is preserved.

The weak Moran’s I for income contrasts with classic class-based theories, suggesting cultural cleavages now dominate U.S. political alignment. The interplay between socioeconomic and cultural factors in voting patterns in the United States, especially during the Trump era, has been a widely discussed topic [23,26,39,40]. Our findings align with research on identity-driven voting, showing religion eclipses income as a predictor of electoral clustering in the Trump era. Religion, and the attitude towards religion, contributes to cultural attitudes and value systems [28] and therefore invariably plays a role in political preferences. Our results show non-geographically adjacent counties sharing the same majority religion have more similar voting patterns than counties in the same income bracket or urbanization level. That is, voters’ behavior during the 2016, 2020, and 2024 elections is more similar to that of their religious peers than to that of their class peers, across the country. These values do not show large time variation across the three presidential elections (Figs 8 and 9). While geographic proximity remains the strongest predictor of voting alignment, shared religious identity emerges as a significant secondary driver of political cohesion.

This analysis lends weight to discussions on cultural and ethnic concerns eclipsing economic considerations during the Trump era [26]. However, broad generalizations must not be made without a more complete analysis taking into account deeper problematics such as voter abstentionism and its role in minority ethnic group vote shares [26]. Still, these results do reinforce the central role cultural concerns have played during Trump’s candidatures, which can be modeled with network structures based on shared traits. These make for a useful tool when studying the dynamics of political behavior.

## Conclusions

We present a method to gauge the similarity of voting patterns in the 2016, 2020 and 2024 by taking into account different adjacency frameworks. We develop this idea through the mathematical and statistical method of spatial autocorrelations and Moran’s I. Apart from the traditional Geographical adjacency, where counties are adjacent if they share a physical border, we further define three alternative types of adjacency: Confessional, Median Household Income and Urbanization level. To construct the necessary adjacency matrices we assign a category to each county and consider counties to neighbor each other if they belong to the same category. This allows us to quantify the degree of similarity of voting percentages between counties that share cultural or socioeconomic characteristics. For the percentage of total votes by candidate in the presidential elections of 2016, 2020 and 2024, we construct the adequate adjacency matrices and calculate the Moran’s I statistic for the following adjacency frameworks: Geographical, First Religion (CHR), First Religion, Second Religion, Third Religion, Fourth Religion, Income and Urbanization.

Defining adjacency in the traditional geographical way yields the highest Moran’s I for all years. Two counties bordering each other remains the best predictor for the similarity in their voting results. However, counties sharing a majority religion (when not grouping together all White Christian groups) exhibit high autocorrelation values that approach those of geographical autocorrelation. Even if geographically far apart, counties sharing a majority religion is a powerful predictor of the similarity in voting patterns. When grouping together all White Christian groups, which is an approximate way the ethnic make up of counties, a lower positive Moran’s I is found in all cases, indicating a high degree of diversity in the voting patterns of White Americans. Socioeconomic frameworks of adjacency like income bracket and urbanization level show lower Moran’s I values, which leads us to conclude that voters have followed their cultural/religious fellows rather than their socioeconomic peers. Non-majority religions in counties are of no use to predict voting pattern similarity.

The fact that geographical adjacency yields the highest Moran’s I across all years is consistent with the enduring importance of direct interpersonal contact in shaping political preferences. Such proximity facilitates local social interactions and informal communication channels, often referred to as “word of mouth”, which remain a relevant and influential mechanism in opinion formation alongside broader cultural and economic factors.

Our results suggest that cultural identity, particularly religion, has played a more powerful role than economic or urban context in electoral behavior during Trump’s candidatures. Counties that share a majority religion exhibit stronger autocorrelation in voting patterns than those grouped by income level or urbanization. This finding raises questions about the assumed primacy of class in politics and points to the relevance of religious and cultural concerns in an era of political polarization. Cultural identity, particularly religion, appears to shape U.S. voting patterns, suggesting that non-economic factors deserve greater attention in political analyses. Sweeping statements must not be made, but the role of culture and religion should not be discarded when analyzing political trends. The topic invites further research into how different dimensions of identity shape political alignment.

## Appendix

S1 Fig and S2 Fig present the spatial distribution of the percentage of votes cast for the Republican and Democratic presidential candidates, respectively, for the 2016, 2020, and 2024 U.S. elections at the county level.

These maps provide the raw electoral landscape underlying our spatial autocorrelation analysis. They visually illustrate the well-known regional polarization in U.S. politics, with large contiguous areas showing consistent partisan preference across multiple election cycles. For each year and candidate, we compute Moran’s *I* using the various adjacency definitions described in the Methods section, including geographical, religious, income, and urbanization criteria.

While the main text discusses the comparative strengths of these adjacency frameworks, presenting the maps here in the Supporting information allows readers to directly relate the quantitative results to the observed spatial patterns in voting. The figures thus serve as a visual foundation for interpreting the clustering measures reported throughout the analysis.

**Expected value and variance of Moran I index.** Moran’s *I* statistic is commonly interpreted through its associated *z*-score, which provides a standardized measure for assessing the significance of observed spatial autocorrelation. This requires knowing the expected value *E*[*I*] and variance *V*[*I*] of Moran’s *I* under the null hypothesis of spatial randomness.

These quantities depend on the number of spatial units *N*, the spatial weight matrix *W*_*ij*_, and the distribution of the observed variable *X*_*i*_. The following expressions present the analytical forms for *E*[*I*] and *V*[*I*] used in this study, following standard derivations in spatial statistics, and are employed to compute the reported *z*_*I*_-scores in our analysis.

The *z*_*I*_-score is defined as:

$$z_{I}=\frac{I-E[I]}{\sqrt{V[I]}}$$ (7)

where *E*[*I*] is the expected value and *V*[*I*] is the variance. The expected value and the variance of Moran’s index are given by:

$$\left\{ \begin{array}{c} E[I]=\frac{-1}{N-1}\\ V[I]=E[I^{2}]-(E[I]{)}^{2} \end{array}\right.$$ (8)

with $E[I^{2}]=\frac{A-B}{C}$ and *A*, *B* and *C* are given by:

$$A=N\left[2\left(N^{2}-3N+3\right)\Sigma_{ij}W_{ij}^{2}-2N\Sigma_{i}{\left(\Sigma_{j}W_{ij}\right)}^{2}+3{\left(\Sigma_{ij}W_{ij}\right)}^{2}\right]$$ (9)

$$B=\frac{2\Sigma_{i}(X_{i}-\bar{X}{)}^{4}}{{\left(\Sigma_{i}(X_{i}-\bar{X}{)}^{2}\right)}^{2}}\left[\left(N^{2}-N\right)\Sigma_{ij}W_{ij}^{2}-2N\Sigma_{i}{\left(\Sigma_{j}W_{ij}\right)}^{2}+3{\left(\Sigma_{ij}W_{ij}\right)}^{2}\right]$$ (10)

$$C=\left(N-1\right)\left(N-2\right)\left(N-3\right){\left(\Sigma_{ij}W_{ij}\right)}^{2}$$ (11)

## Supporting information

S1 Fig Percentage of votes going to the Republican candidate across the 2016, 2020 and 2024 presidential elections. We calculate Moran’s I, using different types of adjacency for these county level data.
(PNG)

S2 Fig Percentage of votes going to the Democratic candidate across the 2016, 2020 and 2024 presidential elections. We calculate Moran’s I, using different types of adjacency for these county-level data.
(PNG)
