# Supplementary figures and images for: Religion or class? Measuring voting clustering on religious and socioeconomic lines in US presidential elections
Source: PLoS One. 2025 Oct 6;20(10):e0331959. doi: 10.1371/journal.pone.0331959 (PMC12500129; doi:10.1371/journal.pone.0331959)

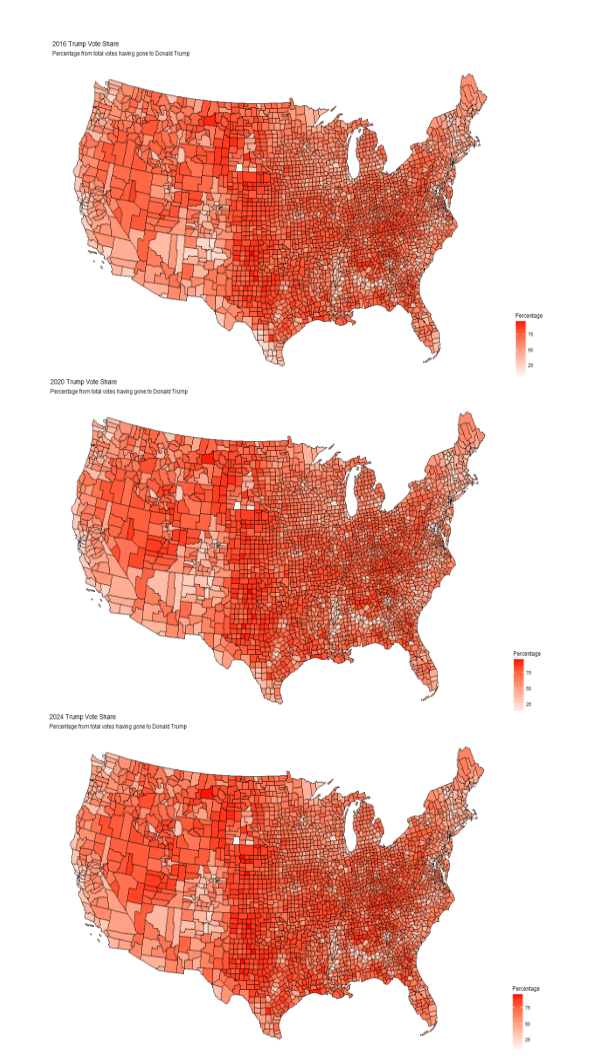

Supplement: S1 Fig — We calculate Moran’s I, using different types of adjacency for these county level data. (PNG) [file pone.0331959.s001.png]

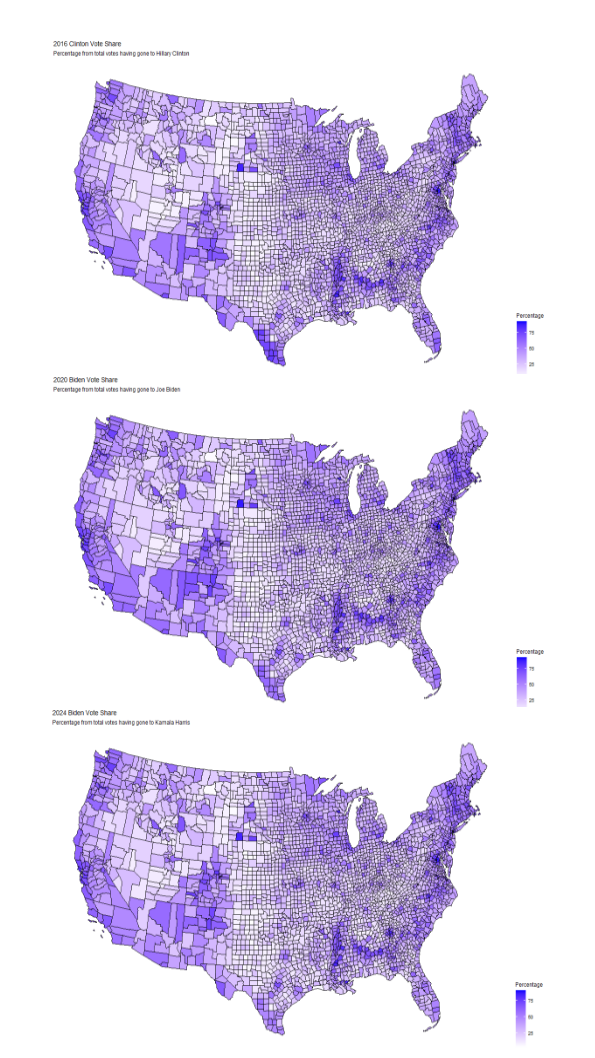

Supplement: S2 Fig — We calculate Moran’s I, using different types of adjacency for these county-level data. (PNG) [file pone.0331959.s002.png]
